# A Novel Soft Biomimetic Microrobot with Two Motion Attitudes

**DOI:** 10.3390/s121216732

**Published:** 2012-12-06

**Authors:** Liwei Shi, Shuxiang Guo, Maoxun Li, Shilian Mao, Nan Xiao, Baofeng Gao, Zhibin Song, Kinji Asaka

**Affiliations:** 1 Faculty of Engineering, Kagawa University, 2217-20 Hayashi-cho, Takamatsu, Kagawa 761-0396, Japan; E-Mails: guo@eng.kagawa-u.ac.jp (S.G.); xiao@eng.kagawa-u.ac.jp (N.X.); gaobaofeng@eng.kagawa-u.ac.jp (B.G.); song@eng.kagawa-u.ac.jp (Z.S.); 2 School of Life Science, Beijing Institute of Technology, Haidian District, Beijing 100081, China; 3 Graduate School of Engineering, Kagawa University, 2217-20 Hayashi-cho, Takamatsu, Kagawa 761-0396, Japan; E-Mails: s12g537@stmail.eng.kagawa-u.ac.jp (M.L.); s11g540@stmail.eng.kagawa-u.ac.jp (S.M.); 4 Kansai Research Institute, AIST, 1-8-31 Midorigaoka, Ikeda, Osaka 563-8577, Japan; E-Mail: asaka-kinji@aist.go.jp

**Keywords:** ionic polymer metal composite (IPMC) actuators, biomimetic underwater microrobot, motion attitudes, micromechanism, shape memory alloy (SMA) actuators

## Abstract

A variety of microrobots have commonly been used in the fields of biomedical engineering and underwater operations during the last few years. Thanks to their compact structure, low driving power, and simple control systems, microrobots can complete a variety of underwater tasks, even in limited spaces. To accomplish our objectives, we previously designed several bio-inspired underwater microrobots with compact structure, flexibility, and multi-functionality, using ionic polymer metal composite (IPMC) actuators. To implement high-position precision for IPMC legs, in the present research, we proposed an electromechanical model of an IPMC actuator and analysed the deformation and actuating force of an equivalent IPMC cantilever beam, which could be used to design biomimetic legs, fingers, or fins for an underwater microrobot. We then evaluated the tip displacement of an IPMC actuator experimentally. The experimental deflections fit the theoretical values very well when the driving frequency was larger than 1 Hz. To realise the necessary multi-functionality for adapting to complex underwater environments, we introduced a walking biomimetic microrobot with two kinds of motion attitudes: a lying state and a standing state. The microrobot uses eleven IPMC actuators to move and two shape memory alloy (SMA) actuators to change its motion attitude. In the lying state, the microrobot implements stick-insect-inspired walking/rotating motion, fish-like swimming motion, horizontal grasping motion, and floating motion. In the standing state, it implements inchworm-inspired crawling motion in two horizontal directions and grasping motion in the vertical direction. We constructed a prototype of this biomimetic microrobot and evaluated its walking, rotating, and floating speeds experimentally. The experimental results indicated that the robot could attain a maximum walking speed of 3.6 mm/s, a maximum rotational speed of 9°/s, and a maximum floating speed of 7.14 mm/s. Obstacle-avoidance and swimming experiments were also carried out to demonstrate its multi-functionality.

## 1. Introduction

Underwater biomimetic microrobots have been extensively employed in various biomedical and naval applications, such as cleaning micro-pipelines in a radioactive environment, submarine sampling and data collection, object recovery in restricted and dangerous spaces, video mapping, scanning blood vessels, and so on [1,2]. In past research, robots have typically utilised motor-actuated screw propellers as actuators. However, the applicability of traditional motors is limited by their large size, high noise, and high power consumption. Because of their electromagnetic configuration, it is difficult to miniaturise motors to fit a compact structure [3,4]. Hence, traditional motors are not a good choice for microrobot design. Because of these problems, smart materials, such as ionic polymer metal composite (IPMC), piezoelectric elements, pneumatic actuators, and shape memory alloy (SMA), are increasingly being applied in microrobotics [5,6]. In particular, SMA and IPMC require relatively low voltages for actuation, and are thus more suitable for compact underwater robots.

Although many biomimetic microrobots with smart actuators have been introduced in recent years, developing a single microrobot with compact structure, flexibility, and multi-functions remains a challenge, due to conflicts between these three characteristics. For this reason, many microrobot designers have abandoned the notion of a compact structure in favour of biomimetic multi-jointed configurations to improve flexibility and obtain multi-functions. Others have sacrificed flexibility and multi-functions in pursuit of miniaturisation. Owing to their compact structure, soft characteristics, low driving power, low noise, operability in water or wet environments, and density similar to that of water, IPMC actuators have been widely researched as a means of actuating microrobots. Since microrobots must make the most of a small volume to realise a variety of functions, smart materials such as IPMC are frequently used as actuators.

The actuation characteristics of IPMC, including suitable response time, high bending deformation, and long life, show significant potential for the propulsion of underwater microrobots. IPMC actuators can be used as undulatory and oscillatory fins to propel swimming microrobots backwards or forwards [7–10], and are widely used for this purpose when fast response is required [8–12]. Typical research in this area has focused on fundamental properties and characteristics, manufacturing techniques, phenomenological modelling of actuation, and sensing mechanisms [13] Yun and Kim [14] proposed a three-fingered gripper in which each finger was an actuator, and each finger was actuated individually. Bonomo *et al.*[15] introduced a nonlinear dynamic model based on a grey box. Branco *et al.*[16] developed a continuum electromechanical model for IPMCs. Porfiri [17] studied the charge dynamics in ionic polymer metal composites (IPMCs) in response to a voltage difference applied across their electrodes. Ahn *et al.*[18] used quantitative feedback theory to implement position control of the IPMC actuator. Gong *et al.*[19] developed a finite element (FE) model for simulating the dynamic electro-mechanical response of an IPMC structure under an external voltage input. But although a large amount of research has been devoted to IPMC-based actuators, deformation and generated force are still under investigation, and a general consensus on the best actuator does not exist [15].

Because the precise position of a fish-like robot cannot be ensured, and its mobility in restricted spaces and capacity for performing some simple underwater tasks are dubious, many researchers have chosen to develop walking robots instead. However, there are still definite areas of inadequacy. Up to now, a single underwater microrobot has only been able to realise a single function of an underwater mission. A ciliary based 8-legged microrobot, for example, implemented only a single walking motion by using IPMC actuators [20]. We need to develop robots that address the shortcoming of unrealised multi-functionality.

We have previously developed several underwater legged microrobots with efficient locomotion capabilities and multi-functionality, employing IPMC-based biomimetic actuators to implement walking, rotating, floating, and swimming motions [21–25]. However, the position precision of the IPMC-actuated legs and fins is not high enough for the performance of some simple tasks, such as detecting an object, grasping and carrying objects to a desired position, or avoiding an obstacle. To implement high-position precision in underwater microrobots, we propose an electromechanical model of an IPMC actuator and analyse the deformation and actuating force of an equivalent IPMC cantilever beam, which could be used to design legs, fingers, or fins for a microrobot. The model is composed of both the electrical and the electromechanical stages, which is simple and accurate to characterize the IPMC actuator. The model parameters can be scaled on the basis of actuator geometry, which is very valuable to investigate the effects of changes in the geometry of the IPMC actuator. This model is accurate enough to estimate relevant deflections of the IPMC actuator when the driving frequency was larger than 1 Hz. For the legs or fins of microrobots are usually driven with a low frequency, the proposed model is capable of describing their electromechanical behaviours, though he displacement variation with respect to voltage is greatly reduced at a high frequency.

To realise the necessary multi-functionality for adapting to different environments, a hybrid underwater microrobot with two motion attitudes is introduced in this paper. The microrobot uses eleven IPMC actuators to implement three-dimensional underwater motions, and two SMA actuators for attitude change. The robot can change between two attitudes: A lying attitude and a standing attitude. It uses the standing attitude to cross a high, narrow gap, and changes to the lying attitude while walking through a low, wide tunnel. We have constructed a prototype microrobot and carried out a series of experiments to evaluate its performance.

The remainder of this paper is divided into five parts. First, we describe the electromechanical model for an IPMC actuator, including the electrical part, theoretical deflection characteristics, and theoretical force characteristics. Second, we experimentally measure the deflection of a sample IPMC actuator to evaluate the proposed theoretical model, and also measure the deflection of an SMA sample. Third, based on several types of biomimetic locomotion, we introduce the new hybrid microrobot design, including the structural design and motion mechanisms in the two attitudes. Fourth, we discuss the construction of a prototype of this hybrid microrobot, together with a series of experiments to evaluate its walking, rotating, floating, and swimming speeds. Attitude change and obstacle-avoidance experiments are also included. Finally, we present our conclusions.

## 2. IPMC Actuators

### 2.1. Electromechanical Model of an IPMC Actuator

**Figure 1.**
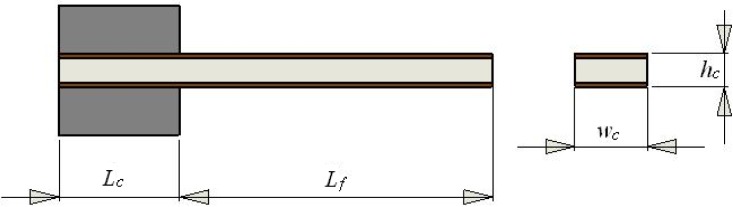
Mechanical configuration of the actuator and relevant parameters.

An IPMC actuator can be represented by an equivalent cantilever beam. Figure 1 shows the mechanical configuration of the actuator and the relevant parameters. Here, *L_c_* denotes the length of the clamped part of the actuator, *L_f_* is the total free length, and *w_c_* and *h_c_* denote the cross-sectional width and height, respectively. The pinned end is used to apply electrical voltages across the thickness.

According to mechanical analysis, the bending deformation of an IPMC actuator results from redistribution of the internal water molecules. Under the influence of an applied stimulus, the water molecules in the actuator are redistributed in the following two stages [19]:

1. When an electrical stimulus is applied across the thickness, each hydrated sodium ion moves in combination with four hydrated water molecules to the cathode side. Bending deformation is generated by the swelling of Nafion 117 near the cathode side, and contraction near the anode side.
2. After a short time, self-diffusion causes free water molecules to flow gradually to the anode side, reducing the concentration of water molecules at the cathode and indicating the deformation recovery potential of the actuator.

Accordingly, the model of an IPMC actuator is divided into two stages. The external stimulus to the model is the applied voltage *V_i_*(*t*), while the first-stage output is an estimate of the absorbed current *I_i_*(*t*). As has been widely reported in the literature, the current produces a mechanical reaction because of the charge/water redistribution [15]. The second stage is intended to estimate either the available force *F*(*t*) or the tip displacement *δ*(*t*) in the absence of an external force.

### 2.2. Electrical Part

Since an IPMC actuator is driven by electrical voltage, it exhibits some electrical characteristics. Thus, we can model the actuator with an equivalent resistance–capacitance (RC) circuit that converts the applied voltage stimulus into an inner current. This RC model is used for determining the electric charge produced by an input voltage. The inner current is in fact a redistribution of the inner ions, and generates an electrical field across the thickness of the actuator. The equivalent RC circuit model with lumped parameters provides some advantages, since it allows a graphical representation of the governing equations for an IPMC leg or finger in an actual application. The electrical elements used in the RC circuit can be evaluated on the basis of physical considerations that enable them to be scaled according to the actuator geometry [15,26–30].

**Figure 2.**
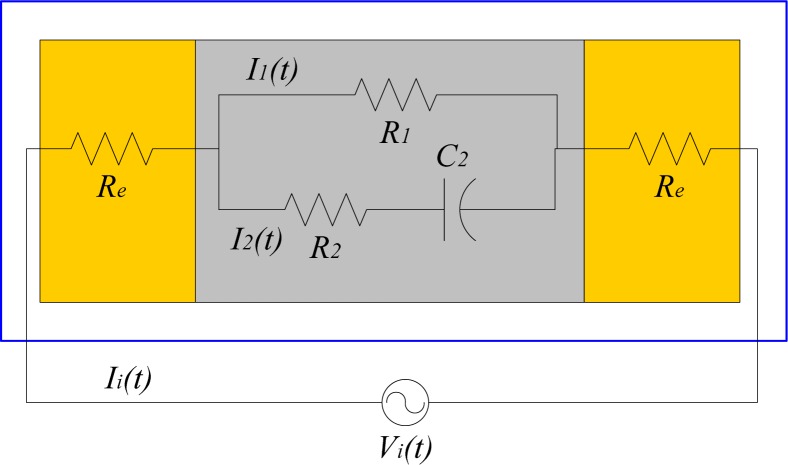
The equivalent electrical circuit for an IPMC actuator.

Figure 2 shows the equivalent lumped RC circuit adopted for the IPMC electrical model. In this circuit, *R*_e_ denotes the electrode resistance, *R*_1_ denotes the equivalent bulk resistance of the Nafion 117, and *R*_2_*C*_2_ reflects the capacitive nature of the IPMC. According to Kirchhoff’s voltage law, we can have:

(1) $$V_{i}\left(t\right)=R_{2}\left[I_{i}\left(t\right)-\frac{V_{i}\left(t\right)-{2R}_{e}I_{i}\left(t\right)}{R_{1}}\right]+{2R}_{e}I_{i}\left(t\right)+\frac{1}{C_{2}}\int_{0}^{t}\left[I_{i}\left(t\right)-\frac{V_{i}\left(t\right)-{2R}_{e}I_{i}\left(t\right)}{R_{1}}\right]\mathrm{dt}$$

where *V_i_*(*t*) denotes the external stimulus, *I_i_*(*t*) denotes the absorbed total current, *I*_1_(*t*) denotes the current across *R*_1_, and *I*_2_(*t*) denotes the current across *R*_2_*C*_2_. It is assumed that there is no initial current flow. We then apply the Laplace transformation to Equation (1), and obtain:

(2) $$V_{i}\left(s\right)=R_{2}\left[I_{i}\left(s\right)-\frac{V_{i}\left(s\right)-{2R}_{e}I_{i}\left(s\right)}{R_{1}}\right]+{2R}_{e}I_{i}\left(s\right)+\frac{1}{{\mathrm{sC}}_{2}}\left[I_{i}\left(s\right)-\frac{V_{i}\left(s\right)-{2R}_{e}I_{i}\left(s\right)}{R_{1}}\right]$$

(3) $$I_{i}\left(s\right)=\frac{s\left(R_{1}C_{2}+R_{2}C_{2}\right)+1}{s\left(R_{1}R_{2}C_{2}+{2R}_{2}R_{e}C_{2}+{2R}_{1}R_{e}C_{2}\right)+R_{1}+{2R}_{e}}V_{i}\left(s\right)$$

The basic charge equation is given by:

(4) $$Q_{i}\left(t\right)=\int_{0}^{t}I_{i}\left(t\right)dt$$

Applying the Laplace transformation to Equation (4) and assuming no initial current flow, we have:

(5) $$Q_{i}\left(s\right)=\frac{I_{i}\left(s\right)}{s}=\frac{s\left(R_{1}C_{2}+R_{2}C_{2}\right)+1}{s^{2}\left(R_{1}R_{2}C_{2}+{2R}_{2}R_{e}C_{2}+{2R}_{1}R_{e}C_{2}\right)+s\left(R_{1}+2R_{e}\right)}V_{i}\left(s\right)$$

### 2.3. Theoretical Deflection Characteristics

The current absorbed by an IPMC actuator induces a mechanical reaction via the redistribution of the inner charges/water molecules, resulting in a mechanical bending of the actuator. The dynamic bending displacement *δ*(*t*) of an IPMC beam is determined by the concentration of water molecules *W*(*t*), as follows:

(6) $$\delta \left(t\right)=k_{v}W\left(t\right)={4k}_{v}Q_{i}\left(t\right)$$

where *k_v_* is the deformation coefficient of the IPMC and *Q_i_*(*t*) denotes the total electric charge. In the saturated state, each sodium ion combines with four water molecules to form a hydrated sodium cation. Hence, *W*(*t*) can be expressed as 4*Q_i_*(*t*). Also, we assume that there is no initial current flow or deformation. We then apply the Laplace transformation to Equation (6), and obtain:

(7) $$\delta \left(s\right)=k_{v}W\left(s\right)=4k_{v}Q_{i}\left(s\right)$$

Substituting *Q_i_*(*s*) from Equation (5), we have:

(8) $$\delta \left(s\right)={4k}_{v}\frac{s\left(R_{1}C_{2}+R_{2}C_{2}\right)+1}{s^{2}\left(R_{1}R_{2}C_{2}+2R_{2}R_{e}C_{2}+2R_{1}R_{e}C_{2}\right)+s\left(R_{1}+2R_{e}\right)}V_{i}\left(s\right)$$

We now scale the elements in the IPMC equivalent circuit according to the following geometrical dimensions: *L_f_* = 17 mm, *L_c_* = 3 mm, *w_c_* = 4 mm, and *h_c_* = 0.22 mm. Since the two electrodes have the same thickness and area, they both have the same resistance, denoted by *R_e_*. By modelling each electrode as a single layer with the same thickness, we can assume that its resistance is proportional to the free length of the actuator and inversely related to its width. *R_e_* can then be determined using the following equation [15]:

(9) $$R_{e}=\frac{R_{s}L_{f}}{w_{c}}$$

where *R_s_* is the induced resistance, which can be estimated via adequate measuring surveys and data processing. Nation^®^ Na^+^ was used in our research. The geometrical dimensions of the IPMC actuator are listed above. Therefore, *R_s_* is approximately equal to 1.075 Ω [15], and the calculated value of *R_e_* is 4.6 Ω. From Equation (9), we can see that the ratio *R_e_*/*R_s_* depends only on the geometrical dimensions of the sample.

*R*_1_ is the equivalent bulk resistance of Nafion^®^ under DC conditions. It can be computed from the following equation [15]:

(10) $$R_{1}=\frac{\rho_{1}h_{c}}{\left(L_{f}+L_{c}\right)w_{c}}$$

where *ρ*_1_ denotes the Nafion^®^ DC resistivity, and *h_c_*, *L_f_*, *L_c_*, and *w_c_* are the geometrical dimensions shown in Figure 1. *R*_1_ can also be obtained from experimental data. For the same IPMC sample, *R*_1_ = 182.1 kΩ.

*R*_2_ denotes the equivalent bulk resistance of Nafion^®^ against the charges involved in fast phenomena. It can be modelled as a function of both the Nafion^®^ resistivity *ρ*_2_ and the geometrical dimensions of the sample, as follows [15]:

(11) $$R_{2}=\frac{\rho_{2}h_{c}}{\left(L_{f}+L_{c}\right)w_{c}}$$

*R*_2_ can also be obtained from experimental data. For the same IPMC sample, *R*_2_ = 0.6523 kΩ.

The capacitor *C*_2_ in the same branch is scaled according to the following Equation:

(12) $$C_{2}=\frac{{ɛ}_{2}\left(L_{f}+L_{c}\right)w_{c}}{h_{c}}$$

The value of the permittivity *ε*_2_ can be determined from the experimental data [15]. For the same IPMC sample, *C*_2_ = 0.04518 F/s.

**Figure 3.**
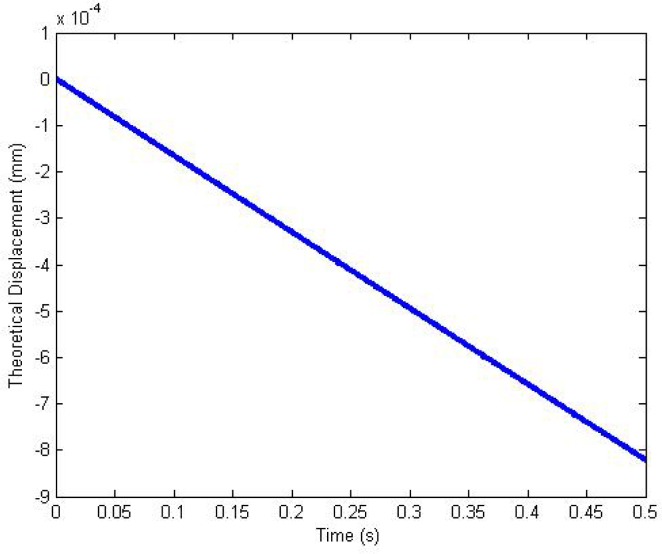
Theoretical deflection of IPMC with time (step stimulus).

The deformation coefficient *k_v_* is assigned a test value approximately equal to 0.06875 for the IPMC sample. We assume an external stimulus *V_i_*(t) = 4(t), so that *V_i_*(s) = 4/*s*. Applying the inverse Laplace transformation to Equation (8), we obtain the following equation:

(13) $$\delta \left(t\right)=0.00166876*\left(\frac{a_{0}-a}{a^{2}}e^{-at}+\frac{a_{0}}{a}t+\frac{a-a_{0}}{a^{2}}\right)=0.0497{\text{e}}^{-0.0334598685t}+6.0403\times 10^{-6}t-0.0497$$

where *a* = 0.0334598685 and *a*_0_ = 0.000121113. The tip deflection of the IPMC sample with respect to time is shown in Figure 3.

### 2.4. Theoretical Force Characteristics

**Figure 4.**
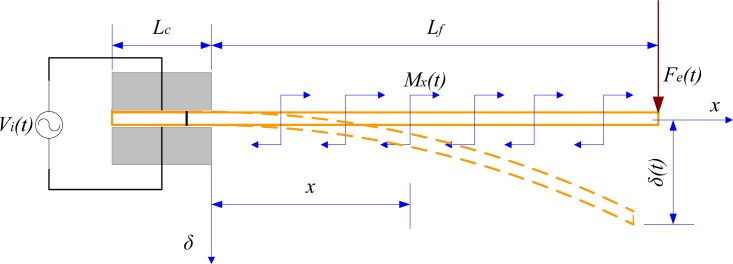
Deflection and distributed bending moment.

Figure 4 shows the electromechanical behaviour of a cantilevered IPMC actuator under an electric field, modelled as a supported cantilever beam under a uniformly distributed bending moment [31]. Utilising the tip deflection equation:

(14) $$\delta_{x}\left(t\right)=\frac{M_{x}\left(t\right)\cdot x}{\mathrm{EI}}\left(L_{f}-\frac{x}{2}\right)$$

under a distributed bending moment, we obtain the equivalent resultant moment at the tip point, given by:

(15) $$M_{l}\left(t\right)=\int_{0}^{l}M_{x}\left(t\right)\mathrm{dx}=F_{e}\left(t\right)\cdot L_{f}$$

substituting *M_l_*(*t*) from Equation (15), we have:

(16) $$\delta \left(t\right)=\int_{0}^{t}\frac{M_{l}\left(t\right)\cdot L_{f}{}^{2}}{2\mathrm{EI}}\mathrm{dt}=\frac{F_{e}\left(t\right)\cdot L_{f}{}^{3}}{3\mathrm{EI}}$$

Applying the Laplace transformation to Equation (16), we obtain:

(17) $$\delta \left(s\right)=\frac{M_{l}\left(s\right)\cdot L_{f}{}^{2}}{s\cdot 2\mathrm{EI}}=\frac{F_{e}\left(s\right)\cdot L_{f}{}^{3}}{3\mathrm{EI}}$$

According to Equation (17), the resultant bending moment and equivalent force at the tip point can be calculated from the following equations:

(18) $$M_{l}\left(s\right)=\frac{s\cdot 2\mathrm{EI}}{L_{f}{}^{2}}\delta \left(s\right)$$

(19) $$F_{e}\left(s\right)=\frac{3\mathrm{EI}}{L_{f}{}^{3}}\delta \left(s\right)$$

We assume an external stimulus *V_i_*(t) = 1(t), so that *V_i_*(s) = 1/*s*. The measured value of the elastic modulus *E* of the IPMC under hydrated conditions is about 83 MPa [32]. For the IPMC cross-sectional dimensions of 0.22 × 4 mm, the moment of inertia *I* of the IPMC is *I* = *w_c_h_c_*^3^/12 = 3.574×10^−15^ m^4^. Applying the inverse Laplace transformation to Equation (19), we have:

(20) $$F_{e}\left(t\right)=0.18114*0.00166876*\left(\frac{a_{0}-a}{a^{2}}e^{-\mathrm{at}}+\frac{a_{0}}{a}t+\frac{a-a_{0}}{a^{2}}\right)=9.0013\times 10^{-3}{\text{e}}^{-0.0334598685t}+1.094\times 10^{-6}t-9.0013\times 10^{-3}$$

## 3. Performance Evaluation

### 3.1. IPMC Actuators

**Figure 5.**
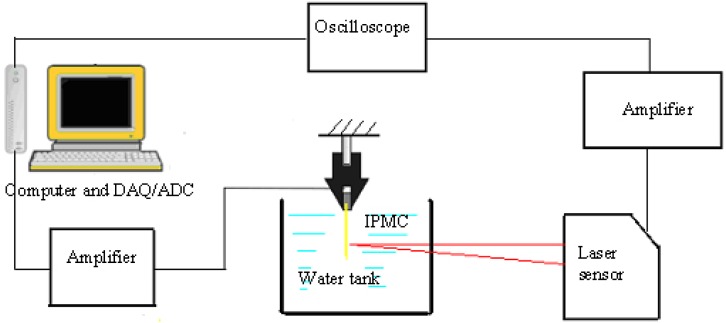
Deflection measurement system for the IPMC actuator.

To evaluate the proposed electromechanical model, we measured the displacement of a single IPMC actuator in a water tank under different applied signals. Figure 5 shows the displacement-measuring system. The actuator was driven by a personal computer (PC) equipped with an analogue-to-digital (AD) converter card, and the deflection of the IPMC was measured by a laser displacement sensor. The laser sensor was used to translate the displacement into a voltage, and the voltages were then recorded and translated to the PC via an oscilloscope. Since the output voltage of the laser sensor is proportional to the distance, we obtained the tip displacement of the IPMC actuator by calculating the change in the voltage. With its capacity for converting a distance signal into a voltage signal, the laser sensor was able to measure the distance at every instant. The IPMC actuator sample was 20 mm long, 4 mm wide, and 0.22 mm thick.

**Figure 6.**
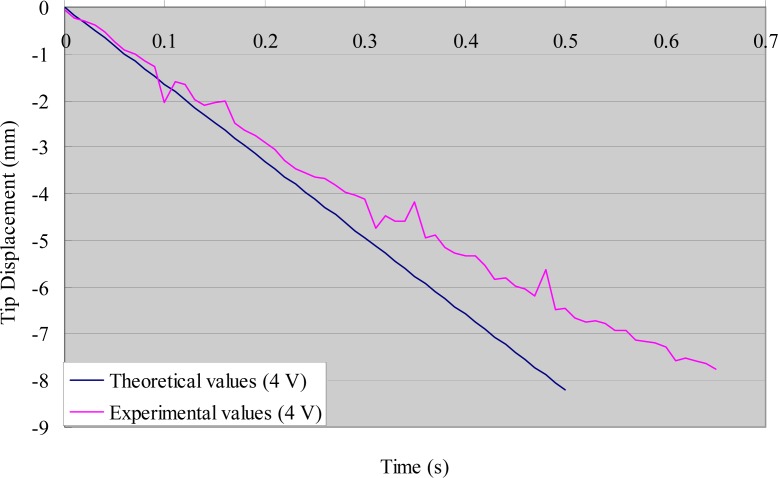
Relationship between the theoretical and experimental values (step stimulus).

**Figure 7.**
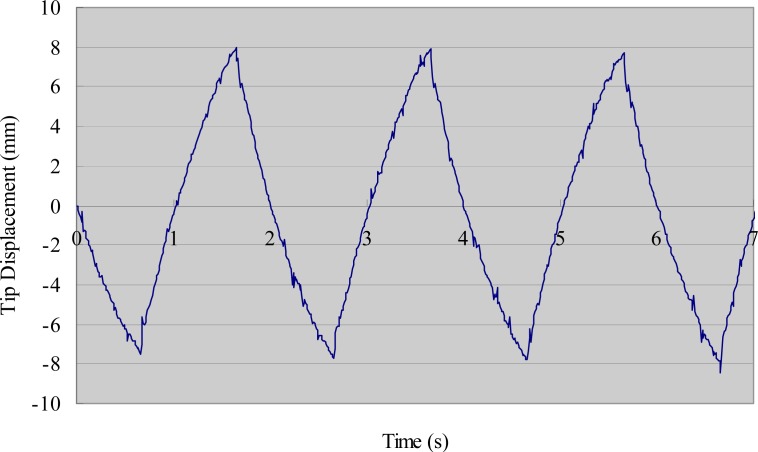
Experimental deflection of IPMC with time (square stimulus).

Figure 6 shows the experimental tip displacements with a step stimulus of 4 V. The theoretical values are included in the figure for comparison. There was good agreement in the first half of the process (0–0.3 s), whereas some errors appeared in the second half (0.3 s onwards). Since IPMC actuators are mainly used as artificial muscles to propel microrobots backwards and forwards, the errors in the second half of the bending process can be ignored when the frequency of the driving voltage is higher than 1 Hz. We measured the deflection of the same IPMC sample with a square stimulus of 4 V, which was used to oscillate the legs in the following section. Figure 7 shows the experimental deflection of the sample with respect to time for a frequency of 0.5 Hz.

**Figure 8.**
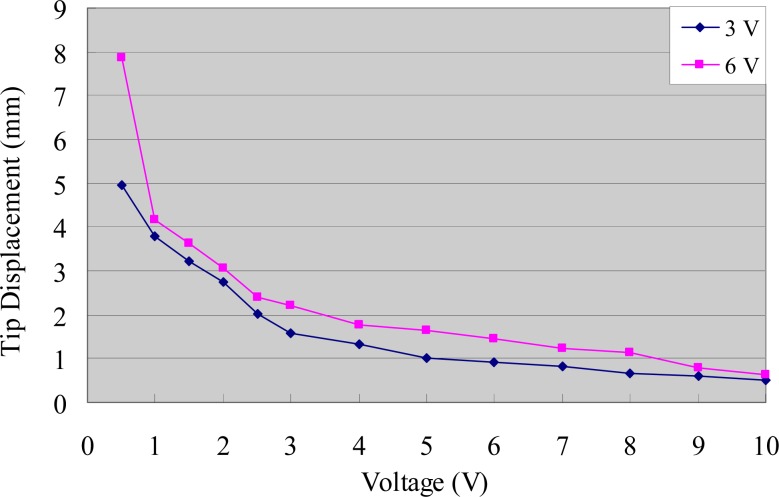
Tip displacements of the IPMC actuator.

The experimental tip displacements of the IPMC sample were measured for different voltages and frequencies. Figure 8 indicates that the displacement was inversely proportional to the frequency of the input signal, and proportional to the input voltage at a low frequency. However, the displacement variation with respect to voltage was greatly reduced at a high frequency.

### 3.2. SMA Actuators

**Figure 9.**
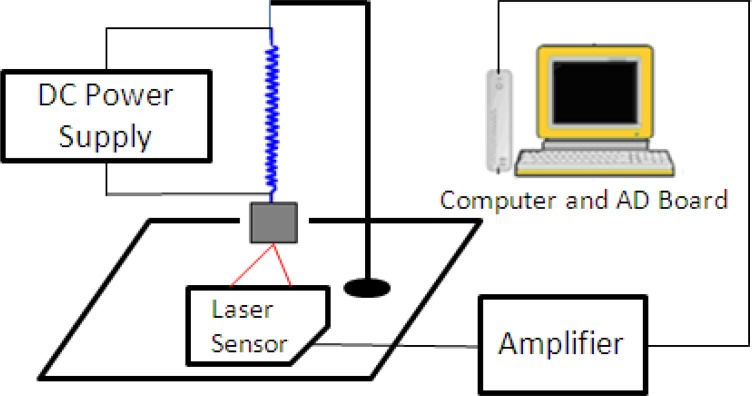
The deformation measuring system.

**Figure 10.**
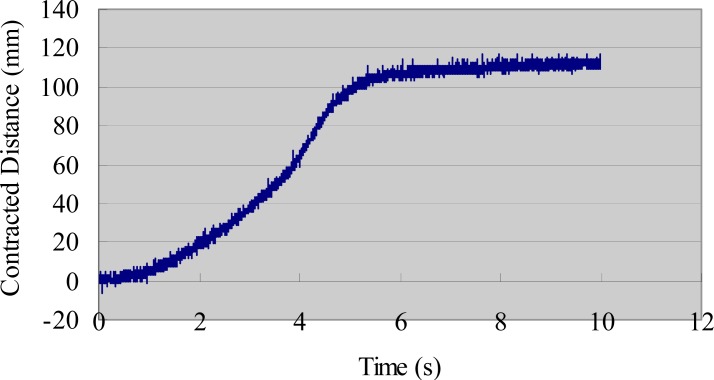
Deformation of the SMA actuator (30 g, 3 V).

**Figure 11.**
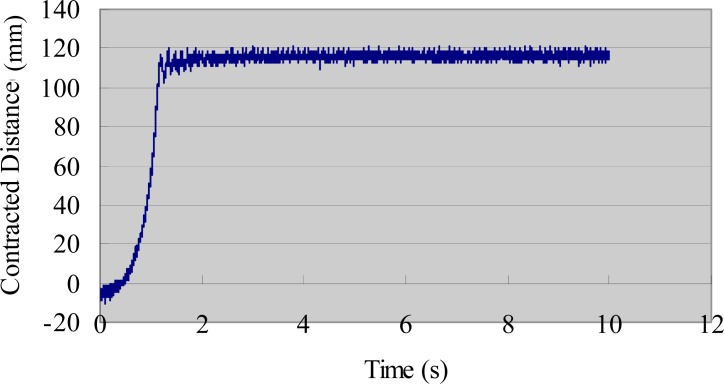
Deformation of the SMA actuator (30 g, 5 V).

**Figure 12.**
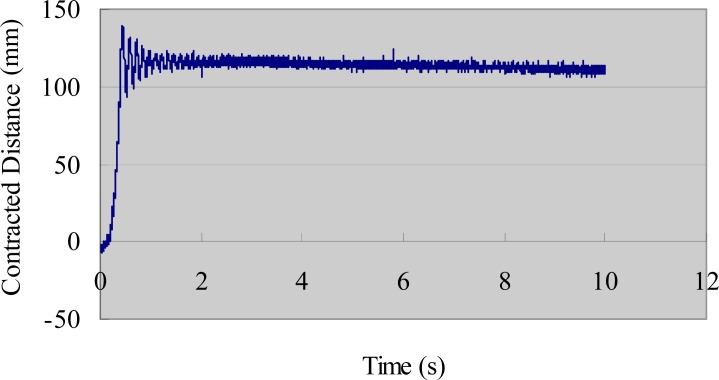
Deformation of the SMA actuator (30 g, 7 V).

We also evaluated the deformation performance of the SMA actuators. We used the same AD board and laser sensor to measure the extended length of the SMA under different input voltages. We measured the deformation of the SMA via the same method used to obtain the tip displacement of the IPMC actuator [33]. Figure 9 shows the deformation-measuring system for the SMA sample. A direct current (DC) power supply provided step input signals to the SMA actuator. The payload weight was 30 g and the testing time was 10 s. The experimental results are shown in Figure 10 (3 V), Figure 11 (5 V), and Figure 12 (7 V). The results indicate that increasing the driving voltage decreased the response time of the SMA actuator. The maximum deformation was almost the same for each of the driving voltages.

## 4. Biomimetic Locomotion and the Proposed Microrobot

### 4.1. Biomimetic Locomotion

Bio-inspired robots borrow their senses and structure from animals, such as insects, fish, and birds. A stick-insect-inspired biomimetic leg prototype using two IPMC actuators was introduced in [21]. The actuator in the vertical direction is called the driver, while the actuator in the horizontal direction is called the supporter. The driver and supporter are driven by two square wave channels, each with the same frequency. The phase of the supporter lags 90 degrees behind that of the driver [21,34].

An inchworm moves by drawing its hind end forward while holding on with its front legs, and then advancing its front end while holding on with its prolegs [23,25]. An inchworm-inspired biomimetic locomotion prototype with two IPMC actuators was introduced to implement fast creeping. The design was based on a one degree-of-freedom (1-DOF) leg. The structure of the 1-DOF walking mechanism is described in [25].

Fish are divided into two categories, based on swimming mode. If a fish generates thrust by bending its body and/or caudal fin, the resulting motion is categorised as body and/or caudal fin (BCF) locomotion. If a fish generates thrust by bending its median and/or paired fin, the resulting motion is categorised as median and/or paired fin (MPF) locomotion [35].

### 4.2. Structure of the Microrobot

**Figure 13.**
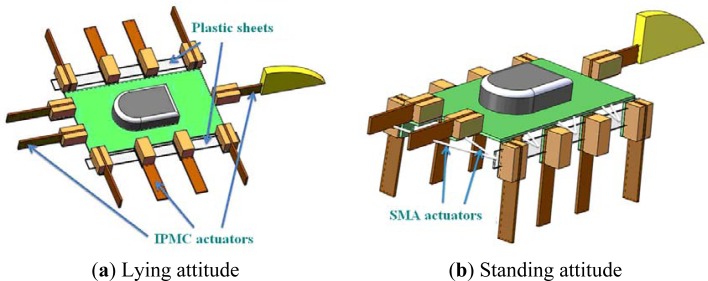
Proposed hybrid microrobot.

Based on the above types of biomimetic locomotion, we propose a hybrid underwater microrobot, consisting of a plastic body, eleven IPMC actuators, two SMA actuators, a passive tail fin, and two plastic sheets. With the SMA actuators affixed to the plastic sheets, the microrobot can change its attitude between the lying state and the standing state, as illustrated in Figure 13. The body of the microrobot is 35 mm long and 20 mm wide, as determined by the motion functions and balance of the overall body. It is 3 mm high in the lying state and 21 mm high in the standing state. The eleven actuators are all 17 mm long, 3 mm wide, and 0.2 mm thick.

**Figure 14.**
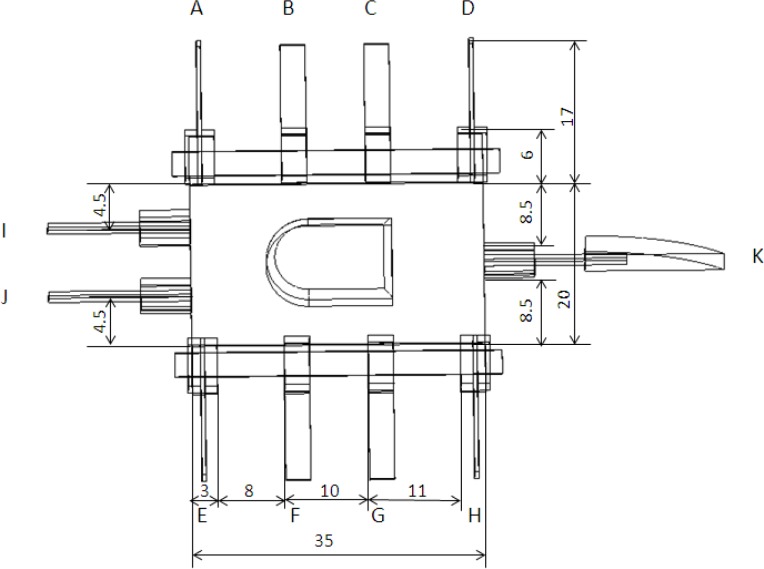
Leg sequence and dimensions of the proposed microrobot.

The microrobot uses eleven 1-DOF IPMC actuators to realise walking, rotating, grasping, swimming, and floating motions [33]. Figure 14 shows the leg sequence of these actuators. In the lying attitude, actuators I and J are used as fingers, and are designed for grasping. Actuators B, C, F, and G are called supporters, while actuators A, D, E, and H are called drivers. By changing the bending directions of the four drivers, the robot can walk forward or backward, and rotate clockwise or counter-clockwise. In the standing attitude, actuators B, C, F, and G are used as fingers for grasping. Legs A and E are used as leading legs, while legs D and H are used as following legs to implement walking and rotating motions. In both attitudes, actuator K is used to actuate the passive tail fin for swimming.

### 4.3. Force Analysis of the Attitude Change

**Figure 15.**
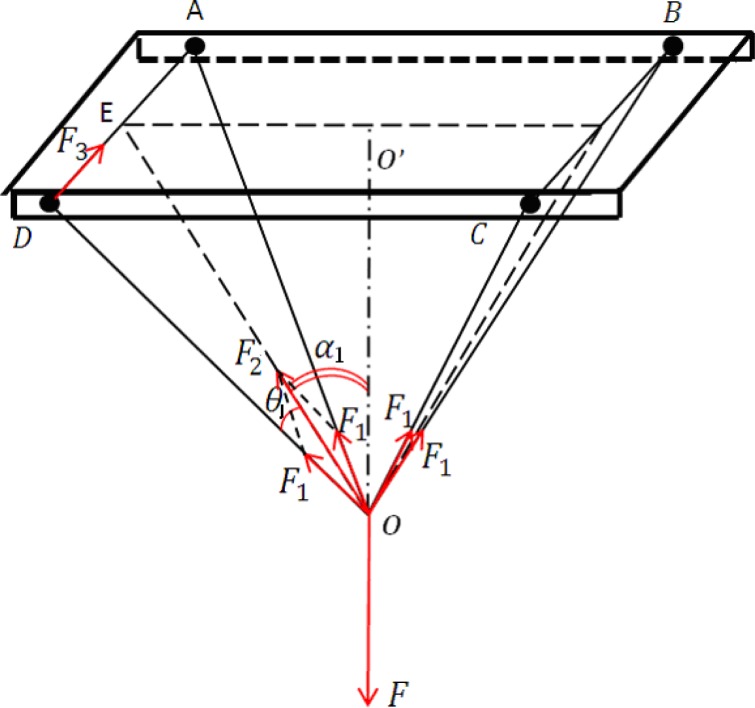
The scheme of tensile force measurement for the attitude change.

The SMA actuators are used to change the attitude of the proposed microrobot. It was necessary to calculate the force required for standing motion before attaching the SMA actuators to the robot body. We then constructed a physical mechanism to transform horizontal forces into vertical forces that could be measured with a spring dynamometer. Figure 15 shows a diagram of the force transition mechanism. We first inserted two fishing lines through the points A–D and B–C, respectively, and then connected the four ends of the two lines at the point *O*. The vertical force *F* was measured via a spring dynamometer at point *O*. The force *F_n_* required to pull the plastic sheet from the horizontal to the vertical direction is given by:

(21) $$F_{n}={2F}_{3}$$

where *F*_3_ denotes the tensile force in either of the lines (AD or BC). According to Figure 15, the tensile forces *F*_3_ and *F*_1_ and the resultant force *F*_2_ can be obtained from the following equations:

(22) $$F_{3}=-F_{1}\text{sin}\theta$$

(23) $$F_{1}=\frac{F_{2}}{2\text{cos}\theta_{1}}$$

(24) $$F_{2}=-\frac{F}{2\text{cos}\alpha }$$

where *F* denotes the measured vertical force.

Utilising Equations (21), (22), (23), and (24), *F_n_* is given by:

(25) $$F_{n}=\frac{F\text{sin}\theta_{1}}{2\text{cos}\theta_{1}\text{cos}\alpha_{1}}$$

We used this formula to calculate the force *F_n_* required for our proposed structure.

### 4.4. Mechanism of the Walking/Rotating Motion in the Lying Attitude

**Figure 16.**
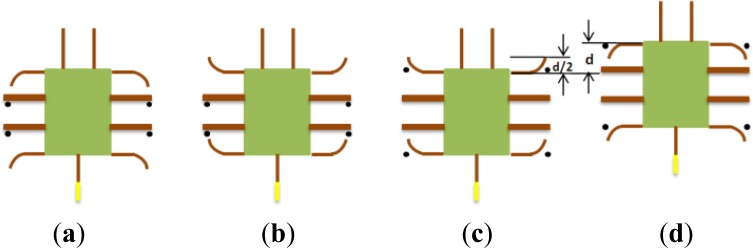
One step cycle of moving forward motion in lying structure (The marks • indicate which actuator contacts the ground).

In the lying attitude, the proposed microrobot can implement stick-insect-inspired walking motions using supporters B, C, F, and G and drivers A, D, E, and H. The drivers provide the propulsion for the motion, and the supporters are employed to raise the drivers off the ground and reduce the resistance. The drivers and supporters are controlled by two square wave channels, each with the same frequency. The phase of the four supporters lags 90 degrees behind that of drivers. Figure 16 shows a single step cycle of the forward motion. Each cycle is divided into four periods as follows [21]:

1. The four supporters lift the body to raise the drivers off the ground.
2. As the supporters lift the body, the drivers bend forward.
3. The four supporters bend upward, causing the four drivers to contact the ground.
4. The four drivers bend backward to push the body forward.

The walking speed is determined by the displacements of the four drivers and the frequency of the control signal. Since the drivers are distributed symmetrically on both sides of the body, and have the same size and deflection characteristics, they bear equivalent loads and drag forces. Therefore, all four drivers provide the same tip displacement for a given applied input voltage. Assuming that the robot is moved by a fixed driving voltage and current, the tip displacement of the actuator in one direction is *d*/2, and the distance the robot advances is *d*, as shown in (Figure 16 (c) and (d)). The walking speed can then be obtained from:

(26) $$v=d\times f=\left(d_{0}-\Delta d\right)\times f$$

where *v* denotes the average walking speed, *d*_0_ denotes the tip displacement of a driver without a payload, Δ*d* is the reduction in the actual displacement of a driver due to friction, and *f* is the frequency of the input signal.

**Figure 17.**
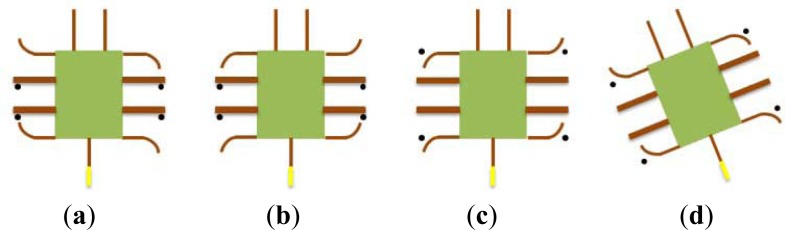
One step cycle of rotating motion in lying structure (The marks • indicate which actuator contacts the ground).

By changing the bending directions of the four drivers, forward and backward walking motions and clockwise and counter-clockwise rotations can be implemented. Figure 17 shows a single step cycle of the rotational motion, which can also be divided into four periods. When the four supporters lift the body, the two left drivers bend forward and the two right drivers bend backward. When the four supporters bend upward, the four drivers contact the ground and bend in the reverse direction.

**Figure 18.**
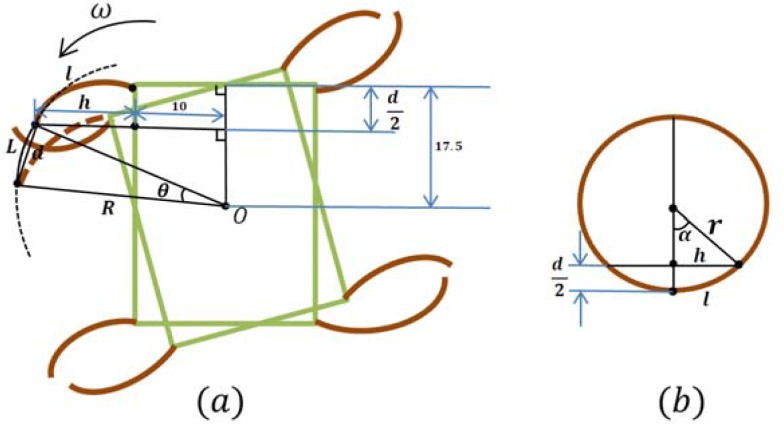
(**a**) The rotating angle in one step cycle. (**b**) The calculation of the value of *h*. (Only drivers are drawn).

When the rotational direction of drivers E and H is opposite to that of drivers A and D, the microrobot can implement clockwise rotation or counter-clockwise rotation. The robot rotates through the angle *θ* in a single step cycle, as shown in (Figure 18(a)). Here, *θ* is given by:

(27) $$\theta =\frac{L}{R}$$

where *L* denotes the length of the rotational arc and *R* denotes the radius of rotation with centre-point *O*. From (Figure 18(b)), we have:

(28) $$r\text{cos}\alpha =r-\frac{d}{2}$$

(29) l=α×r

(30) $$h=\sqrt{\frac{d}{2}\cdot \left|2r-\frac{d}{2}\right|}$$

where *r* is the bending radius of the IPMC actuator, *α* denotes the central angle of the IPMC bending arc, *l* denotes the length of the IPMC actuator, and *h* denotes the semifocal chord length of the IPMC bending arc. The radius *R* can be calculated using the equation:

(31) $$R=\sqrt{{\left(h+10\right)}^{2}+{\left(17.5-\frac{d}{2}\right)}^{2}}$$

when *d* is very small, we can approximate the arc length *L* by *d*, the linear distance between the initial and final robot position. According to Equations (27) and (31), the theoretical rotational speed can then be calculated from:

(32) $$\omega =\theta *f=\frac{2d}{\sqrt{{\left(h+10\right)}^{2}+{\left(17.5-\frac{d}{2}\right)}^{2}}}f$$

### 4.5. Mechanism of the Walking/Rotating Motion in the Standing Attitude

**Figure 19.**
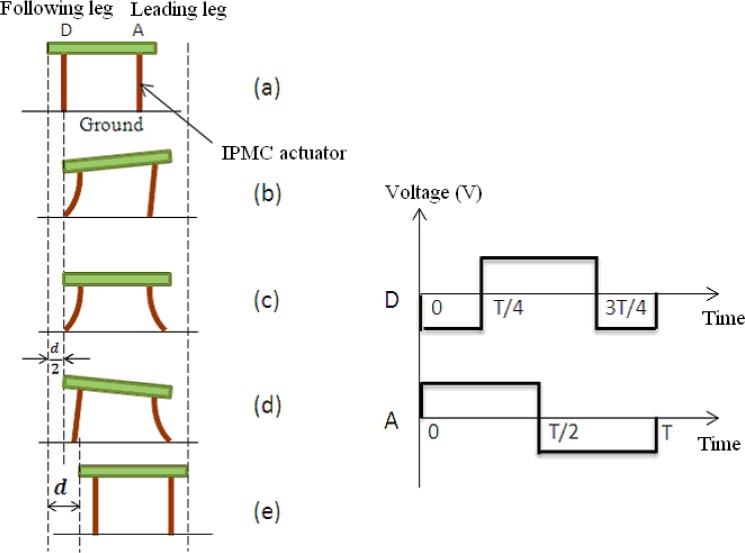
One step cycle of crawling motion in standing attitude [36].

In the standing attitude, the microrobot can implement inchworm-inspired crawling motions in two directions (longitudinal and transverse) using the eight legs A to H. Unlike the motions in the lying attitude, legs A and E are used as leading legs, while legs D and H are used as following legs. This allows the robot to implement walking motion in the longitudinal direction. When the robot walks forward, the phase of the leading leg lags 90° behind that of the following leg, as shown in Figure 19[36]. In this attitude, the robot can fold all legs below its body to get across high narrow gaps. The crawling speed in the standing attitude is determined by the same parameters as in the lying attitude.

Based on this walking mechanism, when one side of the microrobot moves forward and the other side moves backward, or remains stationary, the robot can rotate in either the clockwise or counter-clockwise direction. The rotational speed of the robot is determined by the rotational angle in a single step and the frequency [36].

### 4.6. Mechanism of the Grasping Motion

In the lying attitude, the microrobot can grasp small objects and carry them to a specified location using fingers I and J. First, the microrobot moves close to the object using legs A–H. Second, fingers I and J bend toward each other to grasp the object. Then the microrobot carries the object to the desired destination. In the standing attitude, the microrobot can also grasp small objects using the leg pairs B–F and C–G for this purpose, while legs A, D, E, and H provide the crawling and rotational motions.

### 4.7. Mechanism of the Floating Motion

When the frequency of the driving voltage is decreased to 0.3 Hz, the water around the IPMC actuators is electrolysed. Air bubbles are generated and become attached to the leg surfaces, and the buoyancy of the microrobot is increased. In the lying attitude, four drivers and four supporters are used to electrolyse the water and implement floating motion. In the standing attitude, leg pairs A–E and D–H are used to implement floating motion. The tail fin can also be used to provide buoyancy, and to adjust the balance of the overall body while floating.

### 4.8. Mechanism of the Swimming Motion

In a similar manner to the BCF and MPF locomotion of fish, robots can be classified into body and/or caudal actuator (BCA) types, and median and/or paired actuator (MPA) types [35]. The proposed microrobot utilises the BCA mode, which generates thrust by bending the caudal fin K, as shown in Figure 14. The bending of the caudal fin provides oscillatory motion, and is triggered by a single IPMC actuator. A passive fin is attached to the free end of this actuator to increase the thrust.

## 5. Prototype Microrobot and Experiments

### 5.1. Prototype Microrobot

**Figure 20.**
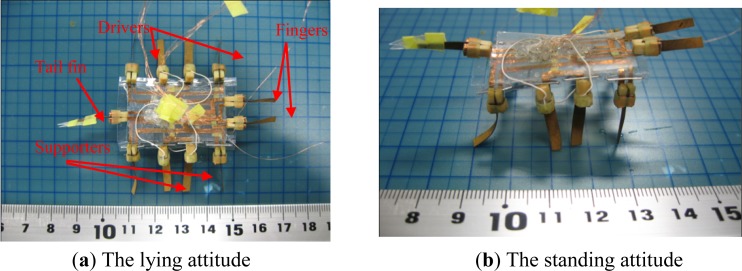
The prototype microrobot (in air).

Based on the proposed structure, a prototype hybrid underwater microrobot with two motion attitudes was constructed, as shown in Figure 20. The body was composed of two layers, to which eleven IPMC actuators were attached with wooden clips. Two IPMC fingers and a tail fin were attached to the first layer, while eight IPMC legs were attached to the second layer. Two SMA actuators were affixed to two sheets attached to the second layer. The prototype microrobot employed eight legs to walk, rotate, and float in two attitudes. Two fingers were utilised to implement grasping, and the tail fin was used for swimming. The control signals of the IPMC actuators were all square waves, in order to drive the actuators more efficiently [36]. In addition, two SMA actuators were employed to pull the two sheets and fold the eight legs below the body, to implement the attitude change. The prototype driving system consisted of an AVR atmega16 and twelve Omron G6K-2P electric relays that were used as circuit changers to vary the input voltages. The microrobot received its control signals through enamel-covered wires with a diameter of 0.03 mm. The wires were soft enough for their resistance to be ignored [21].

### 5.2. Walking, Rotating, and Grasping Experiments in the Lying Attitude on an Underwater Flat

The walking experiments were conducted on a flat underwater surface. In these experiments, we varied the applied signals, and calculated the walking speed by recording the time required to cover a distance of 50 mm. The experiment was repeated five times for each set of control signals to obtain an average speed.

**Figure 21.**
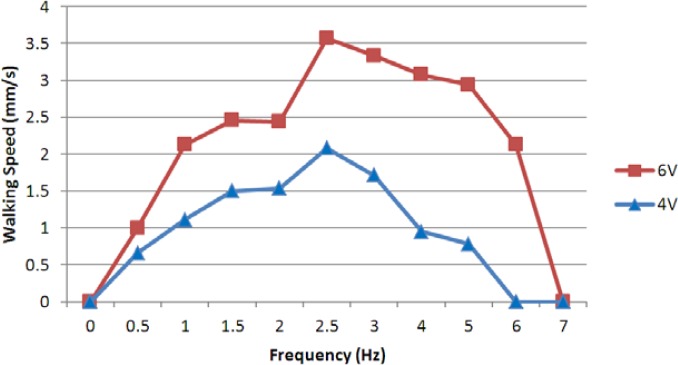
Experimental walking speeds with different frequencies.

At a fixed current of 0.7 A, we carried out two groups of experiments with different applied voltages and frequencies. Figure 21 shows the experimental results for voltages of 4 V and 6 V, which indicated that the walking speed was proportional to the input voltage, and that the walking motion was highly efficient in the control frequency range from 2–6 Hz.

**Figure 22.**
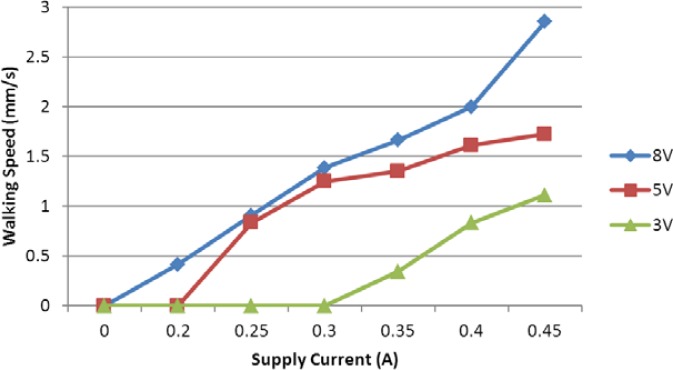
Experimental walking speeds with different currents.

At a fixed frequency of 1 Hz, we also carried out three groups of experiments with applied voltages 3 V, 5 V, and 8 V. We obtained an average speed for every set of signals, varying the current as shown in Figure 22. From the results, the walking speed was proportional to the applied current and input voltage. The microrobot required only low current and voltage for walking motion in the lying attitude.

**Figure 23.**
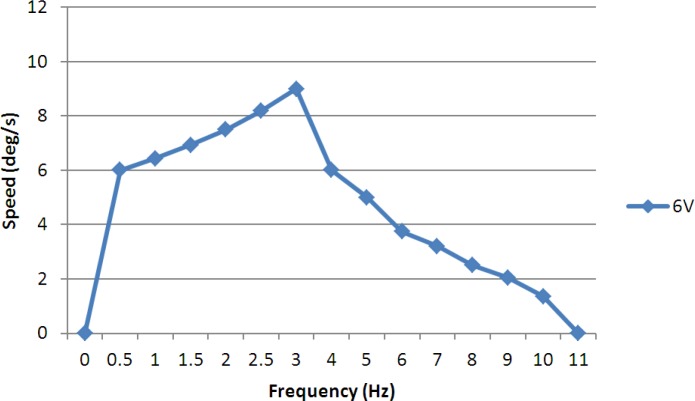
Experimental rotating speeds.

In the rotating experiments, we varied the control frequency from 0.5–11 Hz at a fixed voltage of 6 V and a fixed current of 1 A, and calculated the average rotational speeds. Figure 23 shows the experimental results, which indicated that the microrobot had a higher rotational speed in the frequency range from 0.5–4 Hz, and a maximum rotational speed of 9°/s. When the control frequency was lower than 3 Hz, the rotational speed was proportional to the frequency, since the oscillatory amplitude was relatively large. However, when the control frequency was higher than 3 Hz, the rotational speed was inversely proportional to the frequency, since the rotational angle in a single step cycle became small, and the decreased displacement became a primary factor affecting the rotational speed.

**Figure 24.**
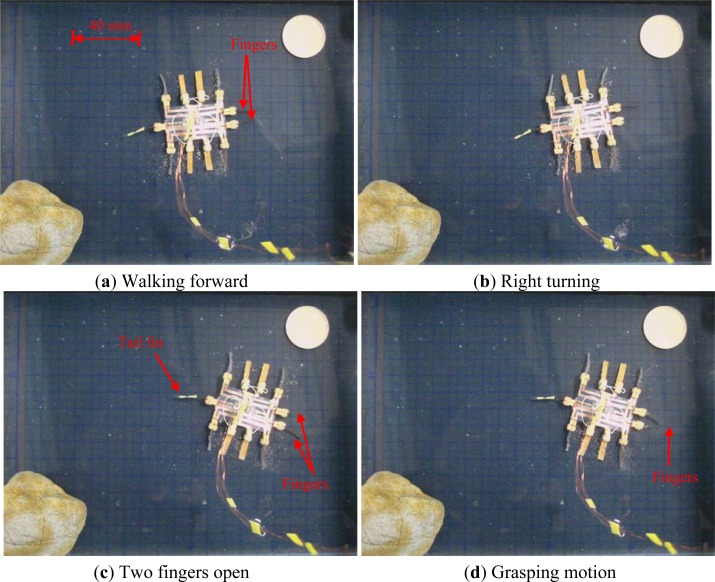

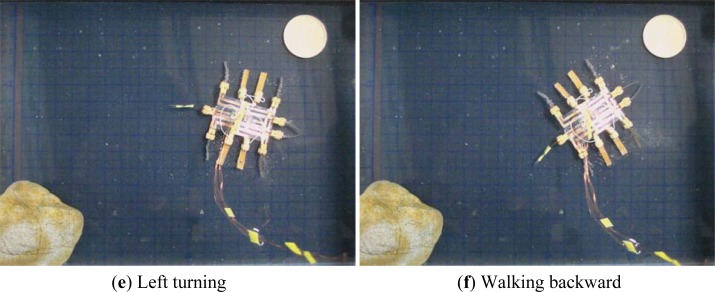
Walking, rotating, and grasping motions.

In the lying attitude, the microrobot was able to use its two fingers to implement grasping motion. A hybrid walking, rotating, and grasping motion is shown in Figure 24. First, the robot walked forward. Second, it rotated clockwise and opened its two fingers. Then it closed its fingers and rotated counter-clockwise. Finally, it walked backward.

### 5.3. Floating Experiments without Payloads

**Figure 25.**
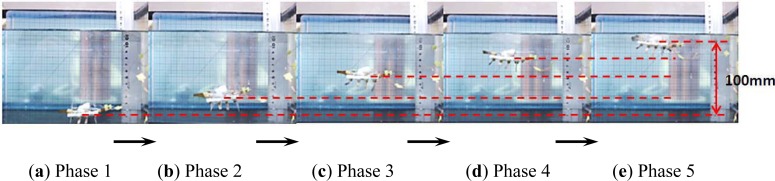
Floating experiment.

Legs A to H were used to electrolyse the water and implement floating motion. In the floating experiments, we varied the frequencies of the driving voltages and calculated the floating speed by recording the time required to float through a vertical distance of 100 mm. Figure 25 shows a video sequence of the floating motion.

**Figure 26.**
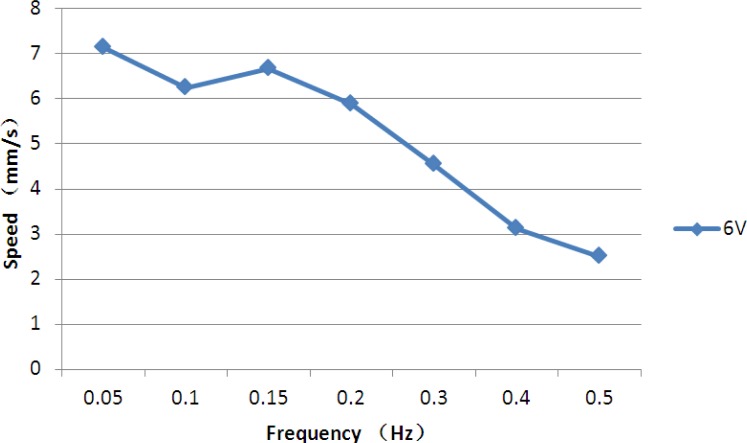
Experimental floating speeds.

At a fixed voltage of 6 V, we varied the control frequencies from 0.05–0.5 Hz. The experiment was repeated five times for each set of control signals to obtain an average speed. Figure 26 shows the experimental floating speeds for different frequencies. From the results, the average floating speed was inversely proportional to the control frequency, and the maximum speed was achieved with a frequency of 0.05 Hz.

### 5.4. Standing Experiments

**Figure 27.**
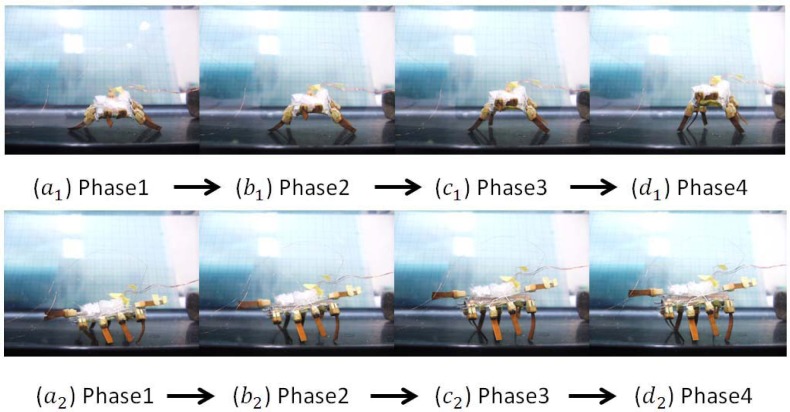
Standing experiments on the underwater flat.

In the standing experiments, we used the two SMA actuators to make the microrobot stand up, both in air and on the underwater flat. Figure 27 shows video sequences of the standing motion on the underwater flat, from the front and left-side perspectives. We carried out the experiments with a control voltage of 8 V and a maximum current of 1 A. An initially deformed SMA actuator can recover its predetermined low-temperature shape during heating, demonstrating the shape memory effect [37]. Therefore, thermal insulation is important for SMA actuators, especially in water. Accordingly, we sealed the two SMA actuators with elastic adhesive tape to achieve a better heating effect when they were triggered to shrink.

### 5.5. Obstacle-Avoidance Experiment

**Figure 28.**
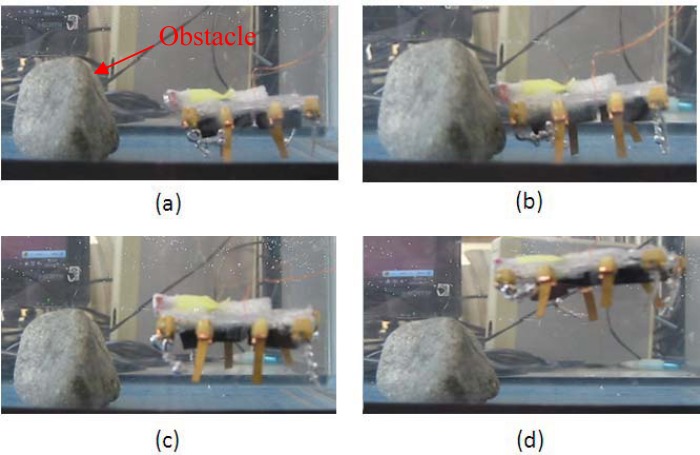
Obstacle avoidance experiment.

To implement closed-loop control, we installed one short-range proximity sensor on the microrobot to detect an object or avoid an obstacle while walking or swimming. The proximity sensor used in the present research was 8 mm long and 5 mm wide, with a weight of 0.5 g. The distance measurement range was 0 to 60 mm, and the output voltage ranged from 150 mV to the power voltage [36]. The sensor signals were transmitted to a micro-AD board, which converted the voltages to digital values and sent them to the AVR. By utilising the proximity sensor, the microrobot was able to detect an obstacle in front of it without any physical contact, and avoided it automatically. In the previous experiments, the microrobot avoided an obstacle by changing its walking direction. However, due to the low rotating efficiency of this unit while in a standing attitude, a long time was required to avoid a very wide obstacle via rotation. Therefore, the hybrid robot avoided the obstacle by floating instead. Figure 28 shows the object-avoidance experiment in the standing attitude. First, the microrobot walked toward the obstacle using legs A, D, E, and H driven by an input voltage of 6 V at a frequency of 1 Hz. When the distance between the microrobot and the obstacle decreased to about 10 mm, the proximity sensor detected the obstacle. The microrobot then stopped and floated upward.

### 5.6. Swimming Experiments in the Standing Attitude

**Figure 29.**
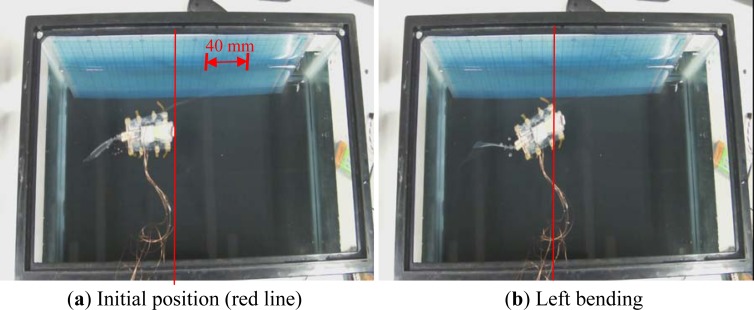

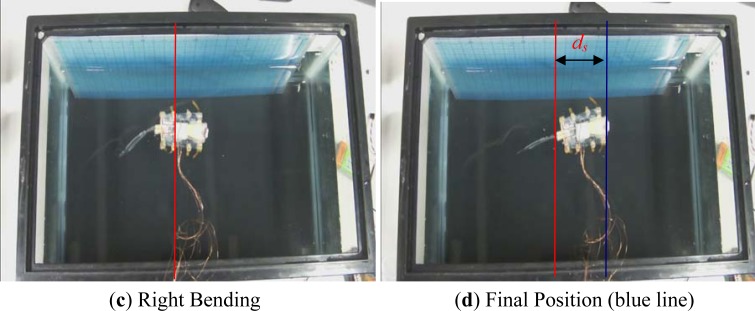
Swimming experiment.

The swimming experiments were carried out in the same water tank. To increase the oscillatory thrust, the swimming motion was evaluated in the standing attitude. Water resistance increases in proportion to the cross-sectional area of the robot body, reducing the oscillatory amplitude of the body. On the other hand, increasing the oscillatory amplitude can reduce the effect of water resistance and increase the swimming speed. The IPMC actuator was actuated by a square wave signal with a frequency of 0.5 Hz and an input voltage of 6 V. The swimming motion for one oscillatory cycle is shown in Figure 29.

## 6. Results and Discussion

Generally speaking, compact structure, multi-functionality, flexibility, and precise positioning are considered incompatible characteristics in underwater microrobots [38,39]. We have already designed several bio-inspired underwater robots with compact structures using IPMC and SMA actuators. These robots employ biomimetic locomotion to implement walking/rotating, surfacing/diving, grasping, and swimming motions. However, each of the units implements only some of these motions. To design a robot with multi-functionality, we need to integrate the above motions in a single robot. There are three types of underwater walking/rotating motions: inchworm-inspired, stick-insect-inspired, and lobster-inspired. Since the position precision of IPMC legs has not been high, in the present research, we proposed an electromechanical model of an IPMC leg for position control. Also, a novel hybrid structure with two motion attitudes was developed to adapt to different environments. Floating can be achieved via the electrolysis characteristics of IPMC, or via jellyfish-inspired or fish-bladder-inspired designs. Since the floating speeds are adjustable in all three of these methods, the first is the best choice to realise a compact structure. Swimming can be achieved via fish-inspired, snake-inspired, butterfly-inspired or manta-ray-inspired designs. However, due to mechanism limitations, only a caudal actuator was suitable for our hybrid design. Accordingly, we used a single IPMC actuator to drive a passive fin in an oscillatory motion. Human-inspired, inchworm-inspired, and lobster-inspired finger locomotion have been proposed for grasping. Our new design not only inherited lobster-inspired finger locomotion, but also implemented inchworm-inspired grasping motion by changing its attitude from lying to standing.

## 7. Conclusions

In this paper, we proposed an electromechanical model for an IPMC actuator to improve the position precision of an IPMC leg, and we introduced a hybrid biomimetic microrobot with two motion attitudes to implement microrobot multi-functionality and flexibility for adaptation to complex underwater environments. In the lying attitude, the new robot implemented stick-insect-inspired walking/rotating motions using eight IPMC legs. These legs were also used to electrolyse the water for floating. Two lobster-inspired IPMC fingers were used to grasp small objects. According to the results of the walking experiments, the robot reached a maximum walking speed of 3.6 mm/s at a control frequency of 2.5 Hz and a fixed current of 0.7 A. The results of the floating experiments indicated that the robot could achieve a maximum floating speed at a control frequency of 0.05 Hz and a control voltage of 6 V. Driven by two SMA actuators, the robot could change its attitude from lying to standing on an underwater flat. In the standing attitude, the microrobot could implement inchworm-inspired walking/rotating using the four outside IPMC legs. The four inside legs were utilised as fingers to grasp large objects. While suspended in the water, the IPMC caudal fin actuated a passive fin to implement oscillatory motion, which provided propulsion for swimming. When equipped with a proximity sensor, the robot could detect and avoid obstacles automatically, either by rotating or by floating.
